# Evaluation of carcinogenic risks related to nitrate exposure in drinking water in Iran

**DOI:** 10.1016/j.mex.2019.07.008

**Published:** 2019-07-24

**Authors:** Mohammad Darvishmotevalli, Maryam Moradnia, Mohammad Noorisepehr, Ali Fatehizadeh, Saeid Fadaei, Hamed Mohammadi, Mehdi Salari, Hamzeh Ali Jamali, Seyede Shahrbanoo Daniali

**Affiliations:** a Department of Environmental Health Engineering, School of Health, Isfahan University of Medical Sciences, Isfahan, Iran; b Department of Health Research Center, Kurdistan University of Medical Sciences, Sanandaj, Iran; c Department of Environmental Health Engineering, Public Health School, Alborz University of Medical Sciences, Alborz, Iran; d Student Research Committee, Faculty of Health, Isfahan University of Medical Sciences, Isfahan, Iran; e Torbat Jam Faculty of Medical Sciences, Torbat jam, Iran; f Department of Environmental Health Engineering, School of Public Health, Hamadan University of Medical Sciences, Hamadan, Iran; g Social Determinants on Health Promotion Research Center, Qazvin University of Medical Sciences, Qazvin, Iran; h Child Growth and Development Research Center, Research Institute for Primordial Prevention of Non-Communicable Disease, Isfahan University of Medical Sciences, Isfahan, Iran

**Keywords:** This research is an comprehensive study which was performed in order to evaluate carcinogenic risks related to nitrate exposure in drinking water in Iran, Cancer risk, Nitrate, Drinking water, Iran provinces

## Abstract

Nitrate is one of the most important contaminants that can release into the environment predominantly as a result of anthropogenic processes. Excessive intake of nitrates may increase the risk of certain types of cancer. The aim of this study was to investigate the concentration of nitrate in drinking water and its health to people in Iran. This cross-sectional study has performed in 2019. Nitrate concentrations in drinking water supplies were obtained from peer-reviewed publications. Monte Carlo stimulations and mathematical models were used to determine the excess cancer risk. Risk level for assessing the carcinogen risk was 10^−5^ (1 per 100,000 persons). Nitrate concentrations and cancer risk related to nitrate were classified by GIS software. According to the obtained results, the drinking water supplies of Tehran, Mashhad (Khorasan Razavi), Zahedan (Sistan and Baluchestan), Shiraz (Fars), Qom, Ardabil and Ahwaz (Khuzestan) have higher nitrate concentrations than the limit recommended by WHO and Institute of Standards and industrial Research of Iran (ISIRI). The estimated cancer risks for the provinces of Tehran, Mashhad (Khorasan Razavi), Zahedan (Sistan and Baluchestan), Shiraz (Fars), Qom, Ardabil and Ahwaz (Khuzestan) were in the no negligible range set by the Health Canada and WHO. The majority of Iran provinces that have impermissible level of nitrate in drinking water supplies had a significant association between cancer prevalence and nitrate exposure.

• The findings demonstrated that carcinogen risk values of nitrate exposure through drinking water was 0.001%.
• Results showed that Tehran, Mashhad (Khorasan Razavi), Zahedan (Sistan and Baluchestan), Shiraz (Fars), Qom, Ardabil and Ahwaz (Khuzestan) are more exposed to additional cancer risk related to nitrosamine.
• The results of this study is considered as the comprehensive report that indicate the association between gastrointestinal cancer and nitrate exposure through drinking water.

## Graphical abstract

**Figure:**
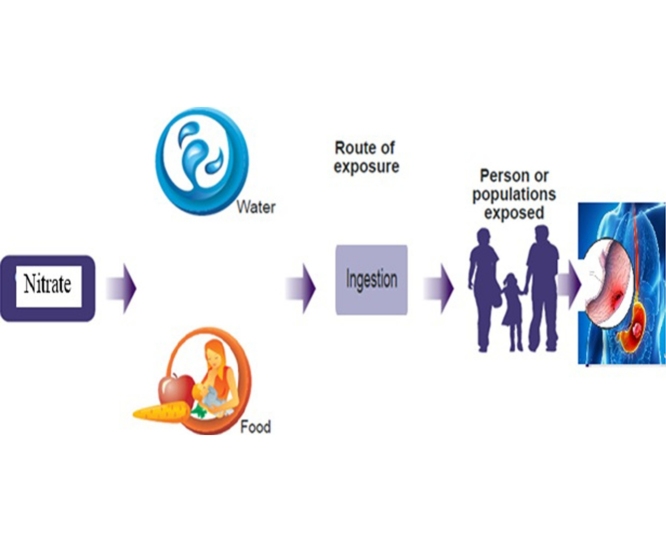

**Specifications Table**

**Table:** 

Subject Area:	Environmental Sciences
More specific subject area:	Cancer risk
Method name:	This research is an comprehensive study which was performed in order to evaluate carcinogenic risks related to nitrate exposure in drinking water in Iran.
Name and reference of original method:	IARC. Ingested nitrate and nitrite and cyanobacterial peptide toxins. IARC monographs on the evaluation of carcinogenic risks to humans. 2010;94 Maryam Moradnia, Mohsen Poursadeghiyan, Amir Hossein Mahvi, Masoud Panahi Fard. The relation of cancer risk with nitrate exposure in drinking water in Iran. Iran J Public Health, Vol. 48, No.2, Feb2019, pp. 362-364.
Resource availability:	The data are available with this article

## Method details

Nitrate and nitrite are naturally produced by biological oxidation of nitrogen in the soil, plants, water, and also lightning. These pollutants are also generally formed by anthropogenic processes such as agricultural activities (including inorganic chemical fertilizer and organic livestock manures), industrial wastewater discharges, wastewater treatment, and motor vehicles [[1], [2], [3]]. Nowadays, intensive usage of nitrogeneos fertilizers in agriculture and runoff are the major pollution source for drinking water supplies [4].

In this study, Monte Carlo analysis and mathematical models [5,6] were used to estimate cancer risk associated with endogenous nitrosamine formation through drinking water containing nitrate concentrations.

Cancer risk associated with endogenous nitrosamine formation is a function of four variables: 1) the amount of nitrite ingested or formed from nitrate, 2) the amount of nitrosatable substances ingested, 3) the rate of *in vivo* nitrosation and 4) the carcinogenic potential of the resulting nitrosamine [7]. According to the above, the modelling techniques were computed from outputs of Monte Carlo simulations are represented as following:

### Estimation of endogenous formation of nitrosamines: exposure model

This model calculates the daily dose of a specific amine formed in vivo as proportional to the amount of ingested amine precursors and the square of the gastric concentration of ingested nitrite using the following equation:

(1) $$DD\;nitros=\;\frac{[{{\text{N}\text{O}}_{2}^{-}]}^{2}\times {DI}_{am}\times \;K_{am}\times 3600\times {MW}_{nitros}}{bw}$$

where DDnitros = daily dose of a specific nitrosamine (mg/kg bw/day); [NO_2_^−^] is gastric nitrite concentration (mol/L), assumed to correspond to the entire amount of salivary nitrite arising from the reduction of the total nitrite from contaminated drinking water by the oral route. This variable is squared because two molecules of nitrite ion are required to form one molecule of the nitrosating species N2O3; DIam is total daily intake of amine (mol/day); Kam nitrosatability rate constant ((mol/L)^−2^ s^−1^), an indication of the relative ease of nitrosation of a specific amine; 3600 is 1 h (measured in seconds); estimation of time during which concentrations of amine and nitrite precursors would remain constant through the oesophageal/cardia region; MWnitros is molecular weight of the specific nitrosamine (mg/mol) and bw is average body weight of an adult, estimated as 70 kg.

### Estimation of gastric nitrite concentrations

(2) $$\left[{\text{N}\text{O}}_{2}^{-}\right]=\;\frac{{[\text{N}\text{O}}_{3}^{-\;}]\times \;\text{T}\text{R}\times \text{W}\text{C}}{{\text{V}}_{\text{S}}}\;$$

where [NO_2_^−^] = gastric nitrite amount (mol/L); [NO_3_^−^] is concentration of nitrate (mol/L) (nitrate concentrations in drinking water supplies (ground water and surface water) obtained from peer-reviewed publications presented in Table 2).

The concentration of nitrate was converted from mg NO3-N/L, via a appointed factor of 4.429 ($\frac{1}{0.226}$ ; 0.226 mg NO3-N/L corresponds to 1 mg/L as NO_3_^−^) and the nitrate molecular weight (62 g/mol); TR is the transformation proportion of nitrate into nitrite, using the highest rate of 0.3; WC is the consumption of water per day (L); and Vs is volume of stomach (L, estimated to be 0.5 L).

### Estimation of daily intake of amines

Specific secondary amine which intakes daily is calculated by multiplying the ingestion rate of each food item by the corresponding amine concentration [8] and summing all foods as follows:

(3) $${DI}_{am}=\;\frac{({{\left[am\right]}_{f}\times {IR}_{f})}_{i}+({{\left[am\right]}_{f}\times {IR}_{f})}_{j}+\ldots +({{\left[am\right]}_{f}\times {IR}_{f})}_{n}\;}{{MW}_{am}}$$

where DI_am_ is the overall intake of a specific amine per day (mol/day); [am]f is amine content in a particular food item (mg/kg); IRf is estimated ingestion amount of particular food item (kg/day), based on Canadian food consumption data [5]; MWam is amine molecular weight (mg/mol); and i, j, …, n are particular food item.

Iranians may be exposed to nitrites and nitrates via various sources including food, drinking water, air and soil. In this study the concentration of nitrite intake through drinking water, the daily dose of a specific nitrosamine and total daily intake of a specific amine through other factors were considered constant.

### Estimation of cancer risk

A cancer risk level present an additional incidence of cancer estimation which might be estimated in an exposed individuals. Shephard et al. applied a non-threshold model for cancer risks estimates, as it represented the worst-case dose–response at low doses. The model assumed that health risk is linearly associated with both the specific nitrosamine which formatted endogenously as daily and the carcinogenic potency [8]. Considering above, in this study, the total cancer risk distribution was predicted by calculating the cancer risk at each individual Monte Carlo exposure result underlying the exposure distribution:

(4) $$\text{E}\text{R}=\;{\text{D}\text{D}}_{\text{n}\text{i}\text{t}\text{r}\text{o}\text{s}}\times \;{\text{C}\text{P}\text{F}}_{\text{h}\text{u}\text{m}\text{a}\text{n}\;}$$

where ER is additional cancer risk related to exposure to a specific nitrosamine dose per day, DDnitros is daily dose of nitrosamine (mg/kg bw/day), calculated in Eq. (1), and CPFhuman is human cancer potency factor ((dose unit/day)^−1^). It is the additional cancer risk related to one daily dose unit of a specified nitrosamine (1.04 × 10^−2^ (μg/kg bw/day)^−1^) [9].

To justify and compare the carcinogen risk assessment related to the contaminated water supplies, several assumptions were used (Table 1). Various guidelines suggested regulation of carcinogens in drinking water at a level which additional cancer risk over a lifetime is essentially negligible. Most regulatory authorities present a risk level of 1 in 100,000 or 1 in 1,000,000. So, in the current research a 10^‐5^ risk level was utilized for the estimation of carcinogen risk.

**Table 1:** Various guidelines and risk Levels.

Authority	risk Levels
U.S. Environmental Protection Agency IRIS Database	10^−6^ cancer risk
Canada proposed Maximum Acceptable Concentration (MAC)	10^−5^ cancer risk
US EPA Regions 3 and 6 non‐enforceable screening level in tap water	10^−5^ cancer risk
U.S. State of California Public Health Goal	10^−6^ cancer risk
World Health Organization Guideline	10^−5^ cancer risk

**Table 2:** The nitrate concentration in drinking water supplies of Iran and the ER related to Nitrate.

NO.	Province’s center	Population	Nitrate concentration (mg/L) and source of water	References	Excess Risk of Cancer	Location in Iran
1	East Azerbaijan	3724620	6- Surface water	[10]	8.6×10^−7^	
2	West Azerbaijan	3080576	17.46- Surface water	[11]	2.5×10^−6^	
3	Ardabil	1248488	57.62- ground water	[12]	1.01×10^−5^	
4	Isfahan	4879312	22.8- Surface water	[13]	3.3×10^−6^	
5	Alborz	2412513	35- Surface water	[14]	5 ×10^−6^	
6	Ilam	557599	10.5- Surface water	[15]	1.5×10^−6^	
7	Boushehr	1032949	5.19- Surface water	[11]	7.5×10^−7^	
8	Tehran	12183391	87.5 - ground water	[16]	2.4×10^−5^	
9	Chaharmahal and Bakhtiari	895263	24.3- Surface water	[17]	3.5×10^−6^	
10	South Khorasan	662534	48.1- ground water	[18]	6.9×10^−6^	
11	Razavi Khorasan	5994402	74.4- ground water	[19]	1.7×10^−5^	
12	North Khorasan	867727	54.9- ground water	[20]	7.8×10^−6^	
13	Khuzestan	4531720	59-Well	[11]	1.1×10^−5^	
14	Zanjan	1015734	24.5- Surface water	[21]	3.5×10^−6^	
15	Semnan	631218	26- Surface water	[11]	3.7×10^−6^	
16	Sistan and Baluchestan	2534327	85- ground water	[21]	4.2×10^−5^	
17	Fars	4596658	72- ground water	[22]	1.6×10^−5^	
18	Qazvin	1201565	20- ground water	[23]	2.9×10^−6^	
19	Qom	1151672	70- ground water	[21]	1×10^−5^	
20	Kurdistan	1493645	21- Surface water	[11]	3.2×10^−6^	
21	Kerman	2938988	20- Surface water	[21]	2.9×10^−6^	
22	Kermanshah	1945227	25.5- ground water	[11]	3.7×10^−6^	
23	Kohgiluyeh and Boyer-Ahmad	658629	20.83- Surface water	[24]	3×10^−6^	
24	Golestan	1777014	23.09- Surface water	[25]	3.3×10^−6^	
25	Gilan	2480874	36.94- Surface water	[26]	5.3×10^−6^	
26	Lorestan	1754243	28- Surface water	[27]	4.1×10^−6^	
27	Mazandaran	3073943	22.6- Surface water	[26]	3.3×10^−6^	
28	Markazi	1413959	47- Well	[21]	6.8×10^−6^	
29	Hormozgan	1578183	46- Well	[21]	6.6×10^−6^	
30	Hamadan	1758268	30.33- Surface water	[28]	4.3×10^−6^	
31	Yazd	1074428	17- Surface water	[29]	2.5×10^−6^	

Nitrate concentrations of water supplies and excess risks related to them were zoned using GIS software for each provincial capital.

The concentration of nitrate in drinking water supplies of Iran provinces and the ER related to that concentration based on their population exposed are presented in Table 2.

According to the obtained results, the drinking water supplies of Tehran, Mashhad (Khorasan Razavi), Zahedan (Sistan and Baluchestan), Shiraz (Fars), Qom, Ardabil and Ahwaz (Khuzestan) have a higher nitrate concentration than the limit recommended by WHO and Institute of Standards and Industrial Research of Iran (ISIRI) [30,31].

**Fig. 1:**
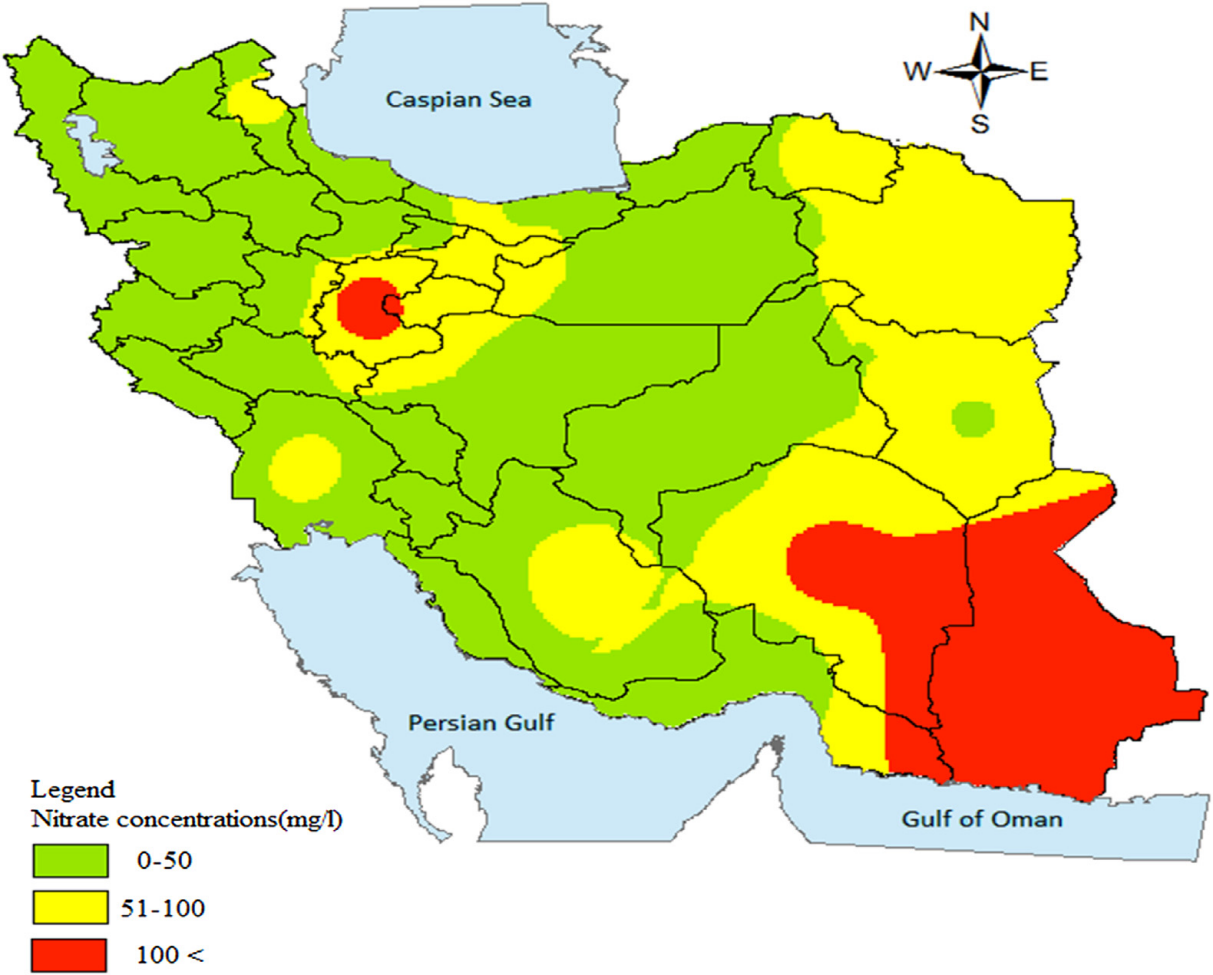
Nitrate concentrations were classified in three group from low to high in Iran.

Nitrate concentrations were classified in three group as high (>100 mg/L as NO_3_), medium (51–100 mg/L as NO_3_) and low (0–50 mg/L as NO_3_) and determined by specific color using GIS software (Fig. 1).

**Fig. 2:**
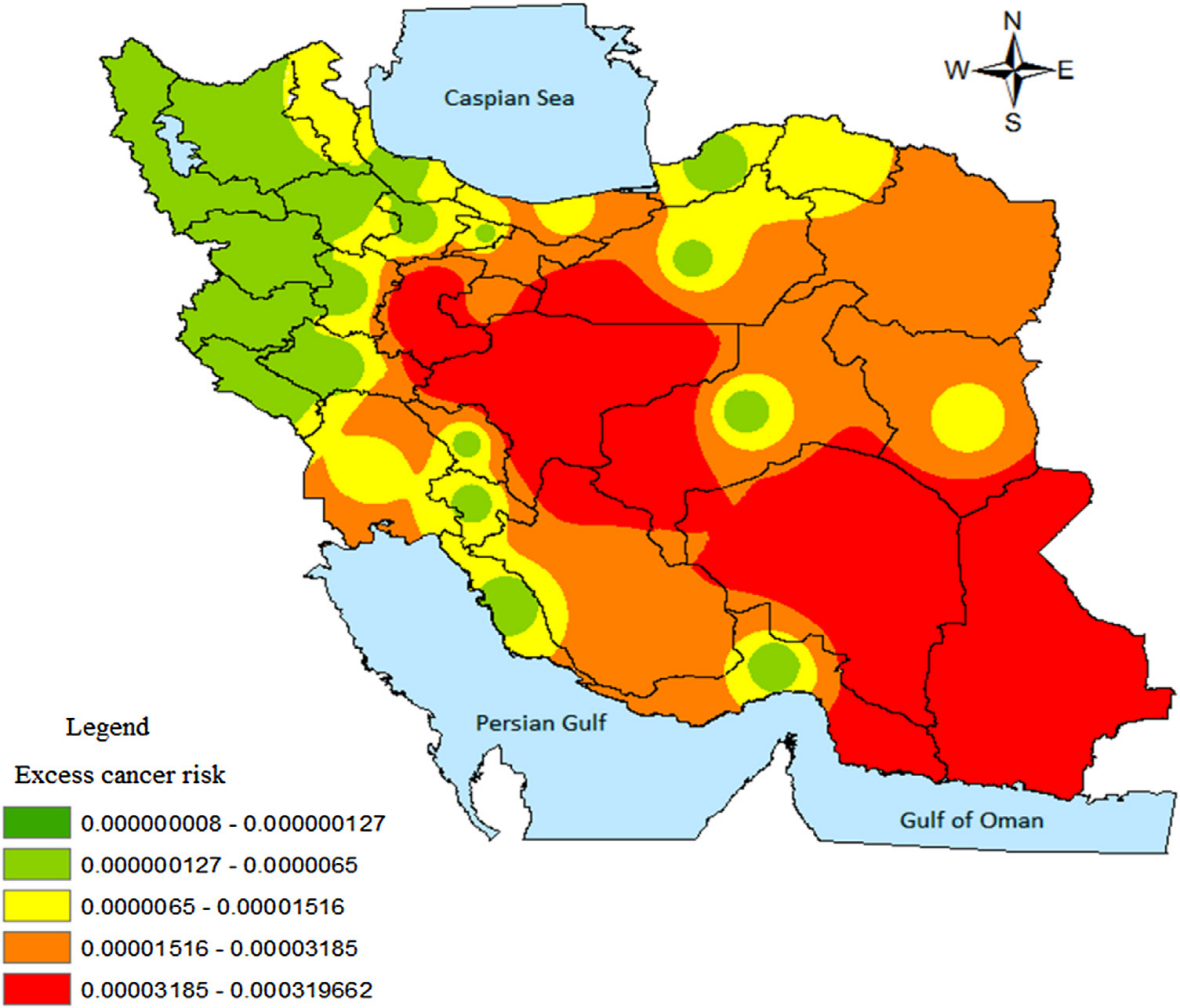
Excess cancer risk related to endogenous nitrosation through drinking water containing nitrate in Iran.

Excess cancer risk related to endogenous nitrosation through drinking water with nitrate was classified in five groups for each province capital which are shown in Fig. 2.

In order to protect human health caused by the potential carcinogenic effects due to exposure of carcinogen N-nitroso compounds, zero concentrations of this contaminant in water is considered based on the non-threshold assumption. Nevertheless, zero value might not be achievable at the present time.

The proposed MAC that has been established for nitrate at 45 mg/L as NO_3_, is protective for the health of the most sensitive subpopulation.

According to risk level of 10^−5^ and the results of Mont Carlo stimulation, the estimated cancer risks for the provinces of Tehran, Mashhad, Razavi Khorasan, Khuzestan, Sistan and Baluchestan, Fars, Qom, and Ardabil are in the no negligible range set by the Health Canada and WHO. The risk of 10^−5^ shows a possibility of one extra case of cancer for every 100,000 persons exposed, the risk of 10^-6^ shows one extra case of cancer for every 1,000,000 individuals exposed. So the probability of extra case of cancer is going up when the number of people exposed increase.

The results of many previous studies in Iran made clear the association between cancer prevalence rates and nitrate exposure through drinking water. It is found that the rate of cancer cases in Fars province increased from 18% to 81% from 1998 to 2005 [32]. According to another study conducted during 1945–1956, the prevalence rates of cancer were 28 in 100,000 persons in south and 42 in 100,000 persons in some north parts of Iran [33]. Furthermore, a series of reports indicated high incidence of gastric cardia adenocarcinoma especially in north and northwestern provinces of Iran especially in Tehran and Ardabil province [11,34] whereas the central and western provinces of Iran were at medium risk and the southern regions were at a low risk [35]. Also the prevalence of non-cardia cancer in Khuzestan, south west of Iran was reported in a high level. According to this report Razavi Khorasan Province in north east of Iran was one of the high risk areas of Iran. In addition an incidence rate of higher than 100 per 100,000 person-years for esophageal cancer has been reported in the north east of Iran [11,26,35,36].

There are many results as mentioned above that were consistent with the obtained results from present study for the northeastern and northwestern provinces including Ardabil, Razavi Khorasan, and Tehran. According to the obtained results from present study, the cancer risk for some central provinces of Iran such as Isfahan and Arak was reported innegligible.

The results of previous study indicated that upper gastrointestinal cancer, especially esophageal cancer was very rare in central part of Iran (for instance the population-based cancer survey in Kerman province of Iran [37]. Also other study indicated a medium risk for these provinces [35]. These results almost are similar to the present study.

Present study showed direct relationship between nitrate exposure through drinking water and the cancer prevalence in southern regions of Iran (Ahwaz and Zahedan) [35].

## Additional information

Nitrate is more commonly found in groundwater than surface water. Most nitrate reduction in soil take place through plant uptake and utilization, while extra nitrates leach into groundwater readily. Groundwater provides drinking water for more than one-half of the world population, and is considered as the single drinking water source for some rural populations and large metropolises [38].

The fate of nitrate in soil depends on the rainwater amount, the water table depth, the organic material content and other physicochemical properties. Extraordinary nitrate amount in drinking water are most often related to private shallow wells and/or depths less than 15 m in districts with permeable soils [39].

High value of nitrate concentrations, up to 500 mg/L have been formerly reported in some groundwater samples in India [40]. In British Columbia, nitrate concentration exceeded 45 mg/L in approximately 60% of 450 well water samples studied in the Fraser Valley. Furthermore, high nitrate level beyond the standard has been reported in various water supplies of provinces of Iran.

The major path of individuals exposure to nitrate/nitrite is consumption of food and drinking water [41]. Short-term exposure to drinking water with a nitrate level at or just above the health standard (10 mg/L as N (equivalent to 45 mg/L measured as NO^−^_3_)) is a potential health problem primarily for infants. Methaemoglobinemia has been considered as the end-point of concern for humans from long-term exposure to nitrate in drinking water. Various documents from animal and human studies recommended that impact on thyroid gland function is an end-point of concern as well. In addition, recent studies has reported relationship between cancer and nitrate exposure in drinking water when conditions result in the formation of nitrosation within the human body [42].

An active endogenous nitrogen cycle in body humans that involves nitrate and nitrite, which are interchangeable in vivo. Nitrosating agents that arise from nitrite under acidic gastric conditions react readily with nitrosatable compounds (especially secondary amines and amides), to generate N-nitroso compounds. These nitrosating compounds are increased following ingestion of additional nitrate, nitrite or nitrosatable compounds. Some of the N-nitroso compounds that could be formed in human body organs under these conditions are known carcinogens [34].

N-Nitrosodimethylamine (NDMA) is a highly water-soluble nitrosamine that is a member of a family of extremely potent carcinogens known as N-nitrosoamines [43,44]. The Integrated Risk Information System (IRIS) of the United States Environmental Protection Agency [45] have categorized some nitrosamines comprising NDMA as possible human carcinogens (B_2_ class) and also as 2A class, probably carcinogenic, by the World Health Organization's International Agency for Research on Cancer [30]. Epidemiological research that evaluated the association between nitrate and cancer have primarily focused on stomach cancer. Consequences from various studies have indicated direct associations, while others have indicated no association and some have shown indirect associations [46,47]. The present study aimed to assess carcinogenic risk of drinking water in difreent provinces of Iran by using Monte Carlo stimulation.

## Conclusions

The results of this study showed that Tehran, Mashhad (Khorasan Razavi), Zahedan (Sistan and Baluchestan), Shiraz (Fars), Qom, Ardabil and Ahwaz (Khuzestan) are more exposed to additional cancer risk related to nitrosamine that formed by nitrate consumed through drinking water. Furthermore, a probability of additional cases of cancer in most populous aera is higher. Also, the findings of the present study also demonstrated that the majority of Iran provinces with impermissible levels of nitrate in drinking water supplies had a significant association between nitrate exposure and cancer prevalence. So, it is important to be concluded that access to safe water is one of essential important factor to decrease the number cancer (especially gastric cancer) prevalence. Finally, some important activities are required for decreasing nitrate concentrations. Some operational technologies for nitrate removal from water supplies include reverse osmosis, ion exchange, electrodialysis and biological denitrification. Furthermore, some protective agents such as dietary antioxidants such as vitamin C are recommended.
